# ﻿*Polypleurumchinense* (Podostemaceae), a new species from Fujian, China, based on morphological and genomic evidence

**DOI:** 10.3897/phytokeys.199.85679

**Published:** 2022-06-13

**Authors:** Bing-Hua Chen, Miao Zhang, Kai Zhao, Xiao-Hui Zhang, Chang-Li Ge

**Affiliations:** 1 College of Life Sciences, Fujian Normal University, Fuzhou 350117, China Fujian Normal University Fuzhou China; 2 The Public Service Platform for Industrialization Development Technology of Marine Biological Medicine and Products of the State Oceanic Administration, Fujian Key Laboratory of Special Marine Bioresource Sustainable Utilization, Southern Institute of Oceanography, College of Life Sciences, Fujian Normal University, Fuzhou 350117, China Fujian Normal University Fuzhou China

**Keywords:** Fujian Province, molecular identification, morphology, Podostemaceae, *Polypleurumchinense*

## Abstract

We describe *Polypleurumchinense*, a new species of Podostemaceae from Yunxiao County, Fujian Province, China, based on morphological and molecular data and the genus *Polypleurum* is recorded here for the first time from China. *Polypleurumchinense* has a gross morphology similar to *P.longistylosum*, but it can be distinguished from the latter by its narrower roots, more numerous and longer leaves, shorter stigmas and more numerous ovules per locule. To distinguish the new *Polypleurum* species and study its phylogenetic position, its complete plastome was sequenced and characterised. The plastome is 132,110 bp in length, including a pair of inverted repeat regions (IRs) of 20,389 bp divided by the large single-copy (LSC) and small single-copy (SSC) regions of 79,022 bp and 12,310 bp, respectively. The plastome size of *P.chinense* is relatively smaller compared to most angiosperms due to the absence of the *ycf*1 and *ycf*2 genes in the IR regions. The phylogenetic analyses also strongly support the separation of the new species from other taxa.

## ﻿Introduction

Podostemaceae, widely known as “river-weeds”, are a vast family of unique haptophtic angiosperms that grow in a variety of wetlands in the tropics and subtropics around the world (Philbrick and Novelo 1995; Cook 1996; Koi et al. 2015). During the wet season, the vegetative phase of Podostemaceae is immersed in rapid and turbulent currents and is tightly adherent to the surface of rocks. As the water level drops over the following dry season, the plants sprout, flower, produce fruit and eventually wither (Tǎng and Kato 2020). During the rainy season, the seeds are spread by wind, birds and running water, the seed coat becomes sticky and sticks to the rock surface, the seeds germinate and the seedlings develop submerged (Koi et al. 2015). Their habitats are unique and are hard to recreate if the water of the stream is contaminated.

Phylogenetically, Podostemaceae are placed in the eudicot order Malpighiales and are sister to Hypericaceae (Wurdack and Davis 2009). Podostemaceae contain 200–280 species in 60 genera, widespread mostly in tropical regions, with a few species in temperate regions. Podostemaceae are divided into three subfamilies, i.e. Podostemoideae, Weddellinoideae and Tristichoideae (Kita and Kato 2001; Koi et al. 2015). Podostemoideae evolved structures and properties obviously unknown in *Hypericum* (Hypericaceae), including loss of apical meristem, reduction or loss of primary shoot and loss of primary root and notably dorsiventral or crustose root and spathella, while Tristichoideae is morphologically similar to Hypericaceae and other terrestrial angiosperms (Koi et al. 2015).

Podostemoideae, the largest subfamily, is divided into various clades, including *Ceratolacis*, *Cipoia*, *Diamantina* & *Podostemum*, American genera (*Apinagia*, *Castelnavia*, *Jenmaniella*, *Marathrum*, *Monostylis*, *Mourera*, *Noveloa*, *Rhyncholacis*, *Wettsteiniola*), *Aulea* (*Saxicolellaproparte*), African genera (*Dicraeanthus*, *Djinga*, *Inversodicraea*, *Ledermanniella*, *Leiothylax*, *Letestuella*, *Macropodiella*, *Monandriella*, *Saxicolellas*.s., *Stonesia*, *Winklerella*), Madagascan genera (*Endocaulos*, *Thelethylax*) and Asian/Australian genera (*Cladopus*, *Farmeria*, *Griffithella*, *Hanseniella*, *Hydrobryum*, *Hydrodiscus*, *Paracladopus*, *Polypleurum*, *Thawatchaia*, *Terniopsis*,*Willisia*, *Zeylanidium*) (Qiu and Philbrick 2003; Koi et al. 2012).

Podostemoideae are the largest subfamily in the family and are widely distributed in Asia, especially in Thailand (42 species with 4 varieties in 10 genera) (Kato 2006; Kato and Koi 2009), India (28 species in 11 genera) (Khanduri et al. 2015), Laos (17 species in 6 genera) (Koi and Kato 2012), Vietnam (7 species in 5 genera) (Kato 2011). In China, nine species in three genera (*Terniopsis*, *Cladopus* and *Hydrobryum*) were reported from Fujian, Guangdong, Hainan, Yunnan, Guizhou, Hong Kong (Kato and Kita 2003; Qiu and Philbrick 2003; Lin et al. 2016; Kato et al. 2017; Zhang et al. 2022).

*Polypleurum* (Taylor ex Tul.) Warm. is a genus previously known from Sri Lanka, India, Thailand and Laos with 17 species (Kato 2006). It is distinguished from *Cladopus* and *Paracladopus* by its more or less flattened, ellipsoid, rough capsule with longitudinal ribs and from *Hanseniella*, *Hydrobryum* and *Thawatchaia* by its ribbon-like root. *Polypleurum* species differ in number of capsule ribs and stamens. Species in Sri Lanka and India, including *P.wallichii* (R. Br. ex Griffz) Warm., the type of the genus, have two stamens and eight capsule ribs, while species in Thailand and Laos have a single stamen and eight to fifteen ribs (Kato 2006).

In January 2021, during a field investigation in Yunxiao County, within the Wushan Mountains in Zhangzhou City, Fujian, China, we unexpectedly discovered a little-known Podostemaceae species during anthesis in a stream. The species has a completely distinct morphology from the other three known genera identified in China. In March and August of 2021, we collected its fruits and vegetative parts for molecular study. Based on morphological analysis and molecular phylogeny, we established that our recently obtained specimen is a new species of *Polypleurum*, a hitherto not recorded genus of Podostemaceae in China.

## ﻿Material and methods

### ﻿Morphological description

The morphological description of the new species was based on the study of specimens collected in a variety of spots in 2021. Live material adhering to rock surfaces from a river in Wushan Mountains, Yunxiao County, Fujian, China, was collected for DNA extraction. A stereoscopic zoom microscope (Carl Zeiss, Axio zoom. v.16, Germany), equipped with an attached digital camera (Axiocam) and a digital caliper were used to record details of roots, leaves, bracts, spathella, tepals, stamen, pistil and seeds. Field observations provided habitats and phenology for the new species.

### ﻿DNA extraction, amplification and sequencing

In this study, total DNA was extracted from freeze-dried material using DNeasy Plant Mini Kit (Qiagen, Valencia, CA, USA). The phylogenetic position of the new species was determined by nrITS and plastid *matK* sequences. The nrITS and plastid *matK* regions were amplified via polymerase chain reaction (PCR) using MiniAmp Thermal Cycler (Applied Biosystems, Foster City, CA, USA) and 1.1xT3 Super PCR Mix (Tsingke Biotechnology, Beijing, China) under the following conditions: 5 min at 94 °C; 30 cycles of 45 s at 94 °C, 45 s at 55 °C, 60 s at 72 °C; and 10 min at 72 °C (Zhou and Jin 2018) and 3 min at 94 °C; 30 cycles of 30 s at 94 °C, 30 s at 55 °C, 90 s at 72 °C; and 7 min at 72 °C (Koi et al. 2012), respectively. The PCR products were treated with Mag-MK 96 Well PCR Products Purification Kit (Sangon Biotech, Shanghai) to remove the extra primers. Sequencing was conducted using the BigDye Terminator v.3.1 Cycle Sequencing Kit (Applied Biosystems) and the ABI 3130xl Genetic Analyser (Applied Biosystems). The primers used for the DNA amplification and the cycle sequencing are listed in Suppl. material 1: Table S1.

### ﻿Genome sequencing, assembly, annotation and analysis

Purified total DNA of *Polypleurumchinense* was fragmented, genome skimming was performed using next-generation sequencing technologies on the Illumina Novaseq 6000 platform with 150 bp paired-end reads and 350 bp insert size by Wuhan Onemore-tech Co. Ltd. (Wuhan, China) and 15.88 GB of reads was obtained.

The paired-end reads were filtered and assembled into complete plastome using GetOrganelle v.1.7.5.0 with appropriate parameters, with K-merset “21,45,65,85,105”, the word size being 0.6 (Jin et al. 2020a). Following previous studies, our workflow includes five key steps as well (Camacho et al. 2009; Bankevich et al. 2012; Langmead and Salzberg 2012; Jin et al. 2020a). Graphs of the final assembly were visualised by Bandage to assess their completeness (Wick et al. 2015). Gene annotation was performed using CPGAVAS2 and PGA. The different annotations of protein coding sequences were confirmed using BLASTx. The tRNAs were checked with tRNAscan-SE v.2.0.3. Final chloroplast genome maps were created using OGDRAW.

### ﻿Phylogenetic analysis

In an attempt to reconstruct the evolutionary history of *Polypleurumchinense*, phylogenies were constructed using Maximum Likelihood (ML) and Bayesian Inference (BI) analyses of the nrITS and *matK* sequences. To construct a phylogenetic tree, based on *matK* sequence, 114 samples (Suppl. material 1: Table S2) of *Terniopsis*, *Cladopus*, *Paracladopus*, *Hanseniella*, *Hydrobryum*, *Hydrodiscus*, *Thawatchaia*, *Hydrobryopsis*, *Zeylanidium*, *Griffithella*, *Polypleurum*, *Willisia* and *Cratoxylum* were included in our analysis. *Cratoxylumcochinchinense* was selected as the outgroup. Each individual sequence was aligned using MAFFT 7.310 (Katoh and Standley 2013) with default settings. A concatenated supermatrix of the two sequences was generated using PhyloSuite v.1.1.15 (Zhang et al. 2019) for the phylogenetic analysis. All missing data were treated as gaps. Gblocks 0.91b (Castresana 2000) was applied to eliminate poorly-aligned regions of the concatenated supermatrix with gaps set as no different from the other positions. The best nucleotide substitution model according to Bayesian Information Criterion (BIC) was TVM+F+R3, which was selected by Model Finder (Kalyaanamoorthy et al. 2017) implemented in IQTREE v.1.6.8. Maximum Likelihood phylogenies were inferred using IQ-TREE (Nguyen et al. 2015) under the model automatically selected by IQ-TREE (‘Auto’ option in IQ-TREE) for 1000 ultrafast (Minh et al. 2013) bootstraps. Bayesian Inference phylogenies were inferred using MrBayes 3.2.6 (Ronquist et al. 2012) under GTR+F+G4 model (2 parallel runs, 2,000,000 generations), in which the initial 25% of sampled data were discarded as burn-in. Phylograms were visualised in iTOL v.5.

To construct a phylogenetic tree based on nrITS, 42 species of *Cladopus*, *Hanseniella*, *Hydrobryum*, *Hydrobryopsis*, *Zeylanidium*, *Griffithella*, *Polypleurum*, *Willisia* and *Cratoxylum* were included in the analysis (Suppl. material 1:Table S3). *Cratoxylumcochinchinense* was employed as the outgroup. The study was carried out as described above and, according to the Bayesian Information Criterion (BIC), the optimal nucleotide substitution model was GTR+F+I+G4.

## ﻿Results

### ﻿Taxonomic treatment

#### ﻿ Polypleurum chinense

Taxon classificationPlantae

B.Hua Chen & Miao Zhang sp. nov.

7DBB4F15-B145-5039-8613-B9AFF82FFAA8

urn:lsid:ipni.org:names:77299427-1

Figs 1 , 2 , 3 , 4

##### Diagnosis.

The new species can be easily distinguished from most other species, except *Polypleurumlongistylosum*, by tufts of leaves on both sides of the root between the root branches, a more or less flattened, ellipsoid, rough capsule with a greater number of longitudinal ribs (> 12), a spathella nearly completely enclosing the ovary and stamen at anthesis, a solitary stamen and a very short capsule stalk (< 2 mm). The narrower roots (0.6–0.8 mm vs.1.0–1.5 mm) with leaves 8–12 per tuft (vs. 4–8), up to 23.1 mm (vs. 5 mm) long, fewer (4 vs. 6) bracts, short spiny or glandular hairs on the spathella (vs. papillate) in the new species differentiate it from *P.longistylosum* (Table 1).

**Table 1.** Morphological differences between *Polypleurumchinense*, *P.longistylosum* and *P.schmidtianum*.

Characteristics	*P.chinense*	*P.longistylosum*	*P.schmidtianum*
Root width/mm	0.6–0.8(−1.0)	1–1.5	2–4
Tufts of leaves position	On both flanks, alternate, subopposite or opposite	On both flanks	Near both sides
The number of leaves	6–12, usually 8–12	4–8	2–4
Leaves length/mm	12.4–23.1	5	1.5–3(−6)
The number and morphology of bracts	4, needle-like	6, needle-like	3–4(−6), sheathed
Bracts length(mm)	5–6	4	2–3
Spathella length/mm	3	-	1.5–2
Spathella coat	Short spiny or glandular hairs	Papillate	Not papillate
Peduncle length/mm	0.7	1	6–7
Tepals length/mm	0.3	0.2	0.5–0.7
Stamen length (mm)	2.9	1.7	1.2
Ovary locular	1	1	2
Ovary length (mm)	1.8	1	1.2–1.5
Stigmas quantity	2, unequal	-	2 or 3
Stigmas length (mm)	0.6–1.2	1–1.2	0.2–0.4
Ovules locule	25–35	10–15	25–35
Capsule stalk length (mm)	1.1–1.6	-	6–12
Capsule ribs	12–14, conspicuous	10–12, inconspicuous	8

##### Type.

China. Fujian Province: Yunxiao County, Wushan Mountains, elevation 430 m, 117°14'E, 23°53'N, 4 January 2021, *Bing-Hua Chen CBH 04407* (Holotype FNU barcode FNU0041131; isotype FNU barcode FNU0041132).

**Figure 1.**
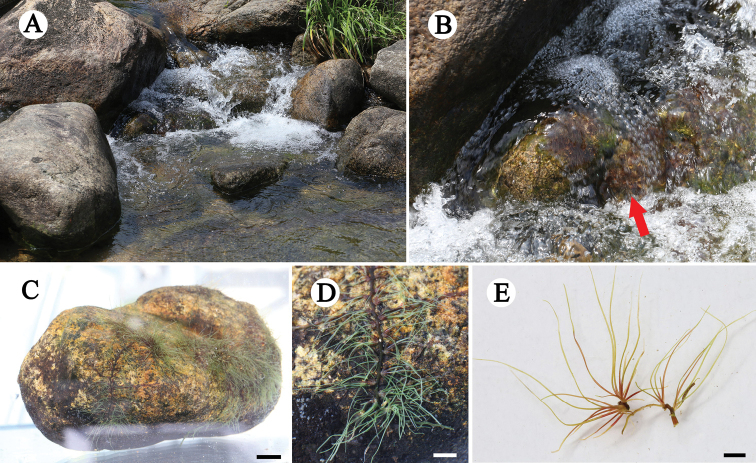
*Polypleurumchinense***A** habitat **B** habit, showing plants (red arrow) on rock surface in rapids **C** plants adherent to rock surface (photo in aquarium) **D** roots with tufts of leaves on both flanks **E** tufts of 1eaves. Scale bars: 5 mm (**C**); 2 mm (**D**); 1 mm (**E**).

**Figure 2.**
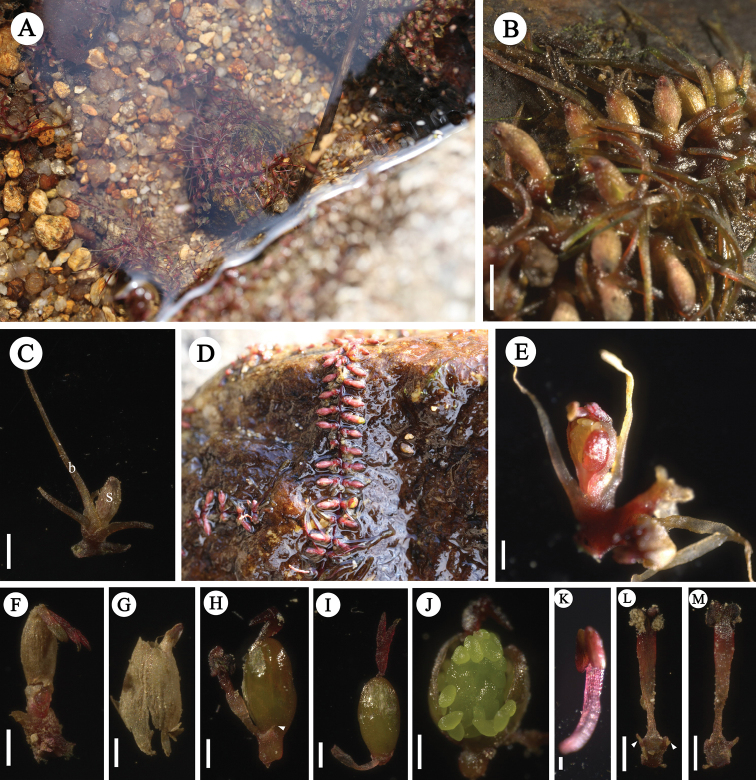
*Polypleurumchinense***A** plants in bud adhering to rock surface **B** ribbon-like root with young floriferous shoots on flanks **C** flower bud covered by spathella(s) above bracts (b) **D** shoots along the flanks of the ribbon-like root between the root branches **E, F** flower at anthesis on peduncle with ruptured spathella **G** spathella **H** flower with spathella removed, stamen, and ovary, arrow shows a tepal on side of stamen **I** gynoecium without bracts **J** ovules on ovary septum **K, L, M** stamen, arrows show two tepals on sides of stamen. Scale bar: 1 mm (**C, F, J**); 2 mm (**B, E, M**); 500 μm (**G, H, I, K, L**).

**Figure 3.**
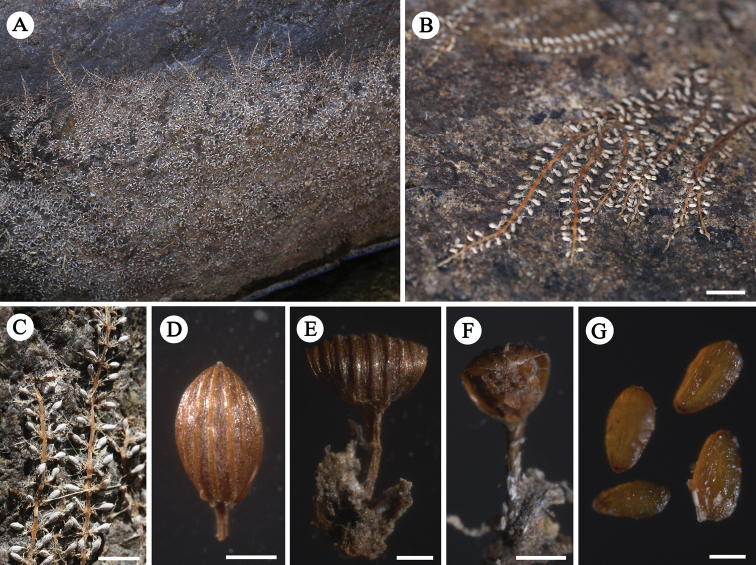
*Polypleurumchinense***A** habitat in the dry season when the river level is reduced **B** habitat showing mature fruits **C** elliptic pale bodies along the flank of the root **D** stalked fruit, showing ribs **E** dehisced capsule, showing seven ribs on the half of capsule, conspicuous **F** dehisced capsule, showing persistent valve and seeds **G** seeds. Scale bars: 20 mm (**B**); 5 mm (**C**); 500 μm (**D**); 400 μm (**E**); 1 mm (**F**); 200 μm (**G**).

Root creeping, adhering to rock surfaces, ribbon-like, branched, 0.6–0.8 (−1.0) mm wide, with tufts of leaves on both flanks, not associated with root branching, 2–4 mm apart; leaves 6–12 per tuft, in two ranks, to 17.6 (12.4–23.1) mm long, 0.2–0.4 mm wide, needle-like (Fig. 1). Flowering shoots on both flanks of root, very short; bracts 2–6, needle-like, to 5–6 mm long, caduceus. Flower just prior to anthesis, with only two remaining bracts, pale purplish-red. The anthesis begins when the water level is further reduced, the bracts disappeared, but the base remained. Flower1, bud covered by ellipsoid spathella, spathella stalk ca. 3 mm in length, coated with short spiny hairs or glandular hairs, with a papilla-like tip, the papilla ruptured near apex at anthesis, but persisting spathella base keeps the ovary and lower bottom of stigma enclosed. Pedicel ca. 0.7 mm long; tepals 2, one on each side of stamen, linear, ca. 0.3 mm long; stamen 1, up to 2.9 mm long, protruding from spathella; ovary dark green, ellipsoid, ca. 1.8 mm long, 0.9 mm wide, 1-locular, free central placenta; stigmas 2, forked near base, thin, needle-like, ca. 0.9 mm long, as long as, or slightly shorter than ovary, branched at the top, upper part exerted from spathella; ovules on marginal surface of septum, 25–35 per septum (Fig. 2). Post-pollination, the spathella and ovary developed into a pale ellipsoid, ca. 2 mm long, arranged on both sides of the root, the ovary stalk lengthened and developed into pedicels (ca. 0.7 mm). Mature capsule 12–14-ribbed, conspicuous under microscope, fissured longitudinally; seed yellowish-brown, with shallow groove, ca. 300 μm long (Fig. 3).

##### Distribution and habitat.

*Polypleurumchinense* is only known from Fujian, China (Fig. 5), where it grows on rocks in unpolluted streams. In addition, *Cladopusaustrosinensis* M. Kato & Y. Kita from the same family was found on the rock surfaces in the lower reaches of the stream. Many other plants grow in the surrounding habitat, whose tree layer includes *Pinusmassoniana* Lamb. (Pinaceae), *Ficusfistulosa* Reinw. ex Bl. (Moraceae), *Caseariaglomerata* Roxb. (Salicaceae), *Caralliabrachiata* (Lour.) Merr. (Rhizophoraceae) and planted Eucalyptusgrandis×urophylla (Myrtaceae) and others; the shrub layer includes *Ficuspyriformis* Hook. & Arn. (Moraceae), *Illiciumdunnianum* Tutch. (Schisandraceae); the vegetation layer includes woody vine plants *Melodinussuaveolens* (Hance) Champ. ex Benth. (Apocynaceae), *Dendrotrophevarians* (Blume) Miquel (Santalaceae), *Toddaliaasiatica* (L.) Lam. (Rutaceae), *Mappianthusiodoides* Hand.-Mazz. (Icacinaceae), *Byttneriagrandifolia* Candolle (Malvaceae), *Uvariaboniana* Finet & Gagnep. (Annonaceae) and more; the herbaceous layer includes *Arundinagraminifolia* (D. Don) Hochr. (Orchidaceae); on the cliff, there are *Cryptochilusroseus* (Lindley) S. C. Chen & J. J. Wood, *Pholidotachinensis* Lindl., *Dendroliriumlasiopetalum* (Willdenow) S. C. Chen & J. J. Wood and other Orchidaceae.

**Figure 4.**
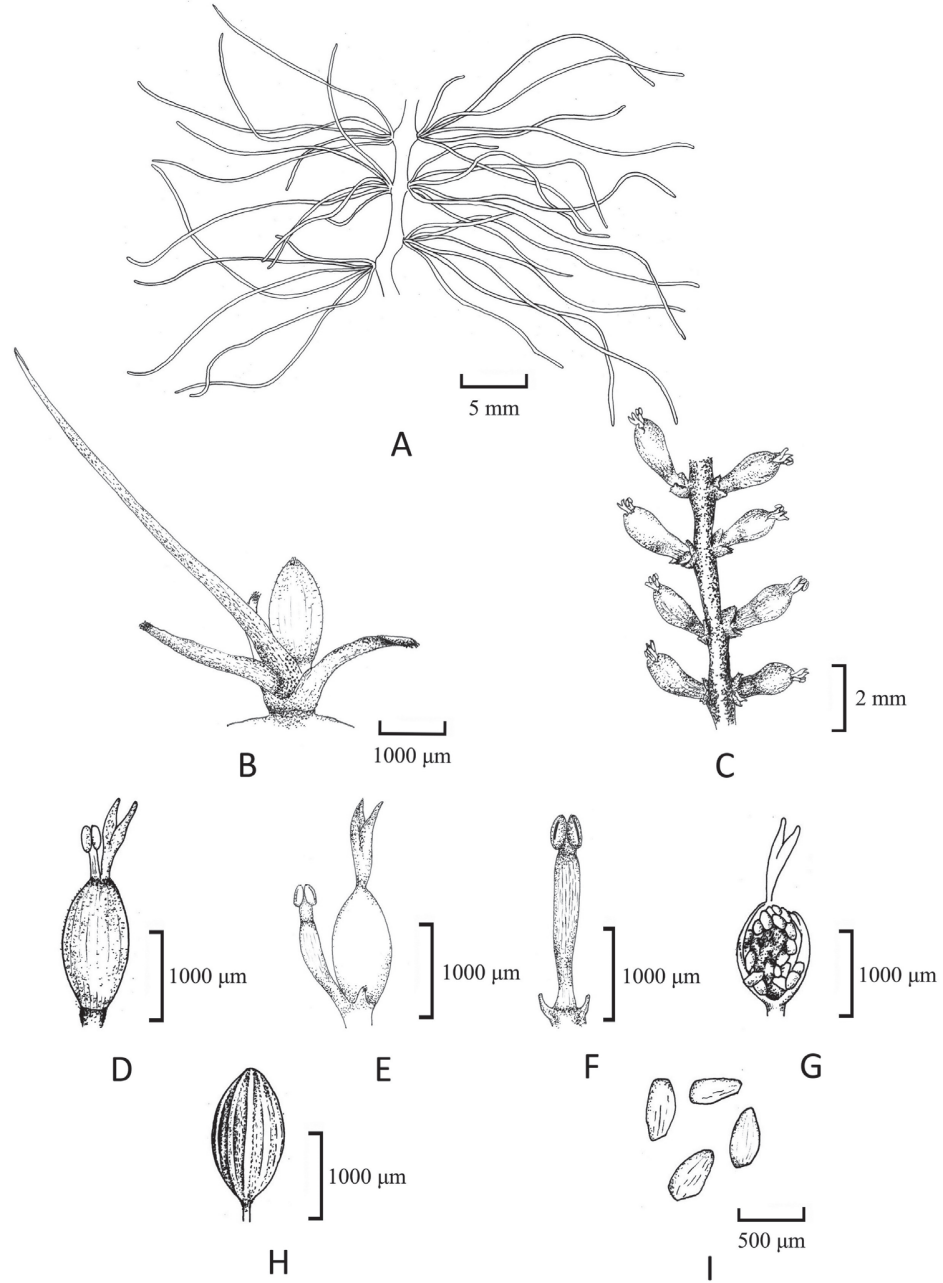
Illustration of *Polypleurumchinense***A** ribbon-like root with tufts of leaves **B** flower bud covered by spathella above bracts **C** floriferous shoots along the flanks of the root between the root branches **D** flower at anthesis on peduncle with ruptured spathella **E** flower with spathella removed, stamen and ovary, a tepal on side of stamen **F** stamen **G** ovules on ovary septum **H** stalked fruit, showing ribs **I** seeds.

**Figure 5.**
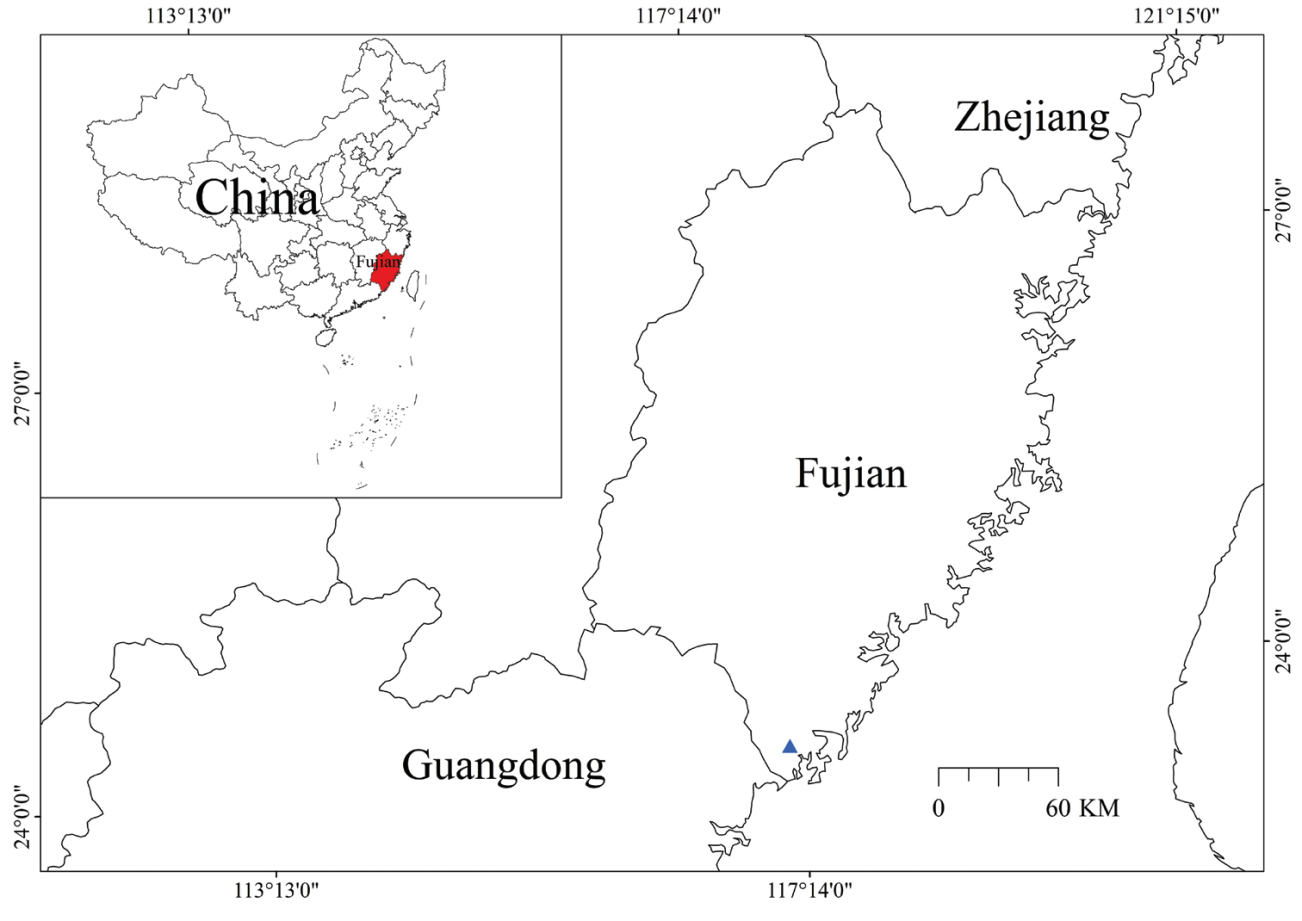
Geographical distribution map of *Polypleurumchinense* (▲). (Map constructed using ArcGis 9.2 software).

##### Phenology.

*Polypleurumchinense* was observed flowering and fruiting in its habitat from December to February when the water level is reduced to partly expose the rocks.

##### Etymology.

The Zhong Guo Cha Pu Chao (中国叉瀑草). The specific epithet “*chinense*” refers to China, as the distribution of this genus was first identified there and it was proven to be a new species of *Polypleurum*.

##### Conservation status.

According to our investigation, *Polypleurumchinense* was found in patches attached to rock surfaces in rapid-flowing streams in the Wushan Mountains range in Zhangzhou City, Fujian Province, China. It is difficult to count the exact number of individuals in the population. Its habitat is vulnerable to anthropogenic destruction and projects like reservoir construction. To determine the exact distribution of this species, further fieldwork is required around the Wushan Mountains in Zhangzhou City and nearby mountainous areas. Therefore, we suggest the species be classified as category DD (Data Deficient), according to the International Union for Conservation of Nature (IUCN 2022). According to the Updated List of National Key Protected Wild Plants (Decree No. 15) by the country’s State Forestry and Grassland Administration and the Ministry of Agriculture and Rural Affairs, all of the known genera of Podostemaceae found in China are classified in the national secondary protection list. The new recorded genus should also be included on the national secondary protection list during the upcoming revision process.

### ﻿Morphology

The new species is morphologically similar to most *Polypleurum* species in that it has ribbon-like roots, tufts of linear leaves on roots, a single flower, a bud covered by spathella and an ellipsoid and rough capsule with longitudinal ribs. However, the new species differs from *P.longtistylosum* and *P.schmidtianum* in the length of leaves, the number of leaves per tuft and capsule ribs, as well as the spathella coat appearance (Table 1).

### ﻿Characteristics of the *Polypleurumchinense* plastome

**Figure 6.**
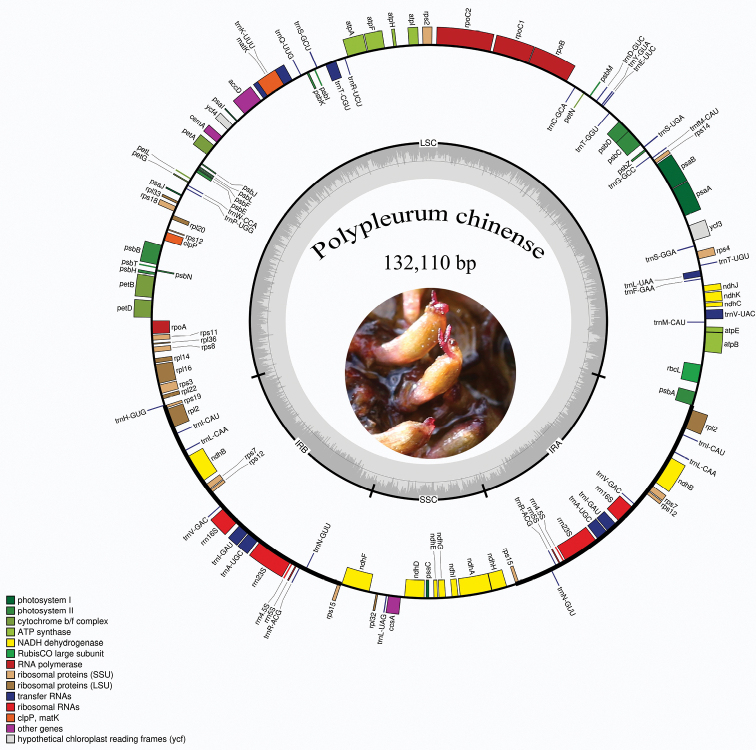
Circular gene map of the plastid genome of *Polypleurumchinense*. Genes inside the circle are transcribed clockwise, while those drawn outside are transcribed counterclockwise. Genes are colour-coded according to their functional groups. The circle inside the GC content graph marks the 50% threshold.

The complete plastome of *Polypleurumchinense* was sequenced and characterised in this study. It is 132,110 bp in length and exhibits a typical quadripartite structure, consisting of a large single copy (LCS) region of 79,022 bp and a small single copy (SSC) region of 12,310 bp, which were separated by a pair of 20,389 bp inverted repeat regions (IRs). The gene map of *P.chinense* plastome is presented in Fig. 6. The gene composition in the plastome of *P.chinense* could be divided into four categories: genes related to photosynthesis, genes related to self-replication, protein-coding genes with unknown functions and other genes. A total of 108 unique genes were identified in the plastome and it contains 72 protein-coding genes, 30 tRNAs, and 4 rRNAs. A total of 16 genes were duplicated in the IR regions, including *ndhB*, *rpl2*, *rps7*, *rps12*, *rps15*, *rrn4.5S*, *rrn5S*, *rrn16S*, *rrn23S*, *trnA*-*UGC*, *trnI*-*GAU*, *trnI*-*CAU*, *trnL*-*CAA*, *trnN*-*GUU*, *trnR*-*ACG* and *trnV*-*GAC* (Table 2). There were four genes lost, including *rpl23*, *infA* and uncommon losses of *ycf1* and *ycf2*. The annotated plastome was documented in GenBank (accession number OL944404).

**Table 2.** Gene contents in the plastid genome of *Polypleurumchinense*.

Category, Group of Genes	Gene Names
**Photosynthesis**:	
Subunits of ATP synthase	*atpA*, *atpB*, *atpE*, atpF*, *atpH*, *atpI*
Subunits of NADH dehydrogenase	ndhA*, ndhB*(*x2*), *ndhC*, *ndhD*, *ndhE*, *ndhF*, *ndhG*, *ndhH*, *ndhI*, *ndhJ*, *ndhK*
Cytochrome b/f complex	*petA*, petB*, petD*, *petG*, *petL*, *petN*
Subunits of photosystem I	*psaA*, *psaB*, *psaC*, *psaI*, *psaJ*
Subunits of photosystem II	*psbA*, *psbB*, *psbC*, *psbD*, *psbE*, *psbF*, *psbH*, *psbI*, *psbK*, *psbJ*, *psbL*, *psbM*, *psbN*, *psbT*, *psbZ*
Large subunit of rubisco	*rbcL*
**Other genes**:	
Subunit of Acetyl-CoA-carboxylase	*accD*
c-type cytochrome synthesis gene	*ccsA*
Envelope membrane protein	*cemA*
Protease	*clpP*
Maturase	*matK*
**Self-replication**:	
Large subunit of ribosome	rpl2*(*x2*), *rpl14*, rpl16*, *rpl20*, *rpl22*, *rpl32*, *rpl33*, *rpl36*
DNA-dependent RNA polymerase	*rpoA*, *rpoB*, rpoC1*, *rpoC2*
Small subunit of ribosome	*rps2*, *rps3*, *rps4*, *rps7* (*x2*), *rps8*, *rps11*, rps12*^a^ (*x2*), *rps14*, *rps15* (*x2*), *rps18*, *rps19*
rRNA Genes	*rrn4*.*5S* (*x2*), *rrn5S* (*x2*), *rrn16S* (*x2*), rrn23S*(*x2*)
tRNA Genes	trnA-UGC*(*x2*), *trnC*-*GCA*, *trnD-GUC*, *trnE-UUC*, *trnF-GAA*, *trnfM-CAU*, *trnG-GCC*, *trnH-GUG*, trnI-GAU*(*x2*), *trnI-CAU* (*x2*), trnK-UUU*, *trnL-CAA* (*x2*), trnL-UAA*, *trnL-UAG*, *trnM-CAU*, *trnN-GUU* (*x2*), *trnP-UGG*, *trnQ-UUG*, *trnR-ACG* (*x2*), *trnR-UCU*, trnS-UGA*, *trnS-GCU*, *trnS-GGA*, *trnT-CGU*, *trnT-GGU*, *trnT-UGU*, *trnV-GAC* (*x2*), trnV-UAC*, *trnW-CCA*, *trnY-GUA*
**Unknown function**:	
Conserved open reading frames	ycf3*, *ycf4*

Note: *genes containing introns; (*x2*) genes present as two copies in the IR regions; ^a^ indicates trans-spliced gene.

### ﻿Phylogenetic analysis

**Figure 7.**
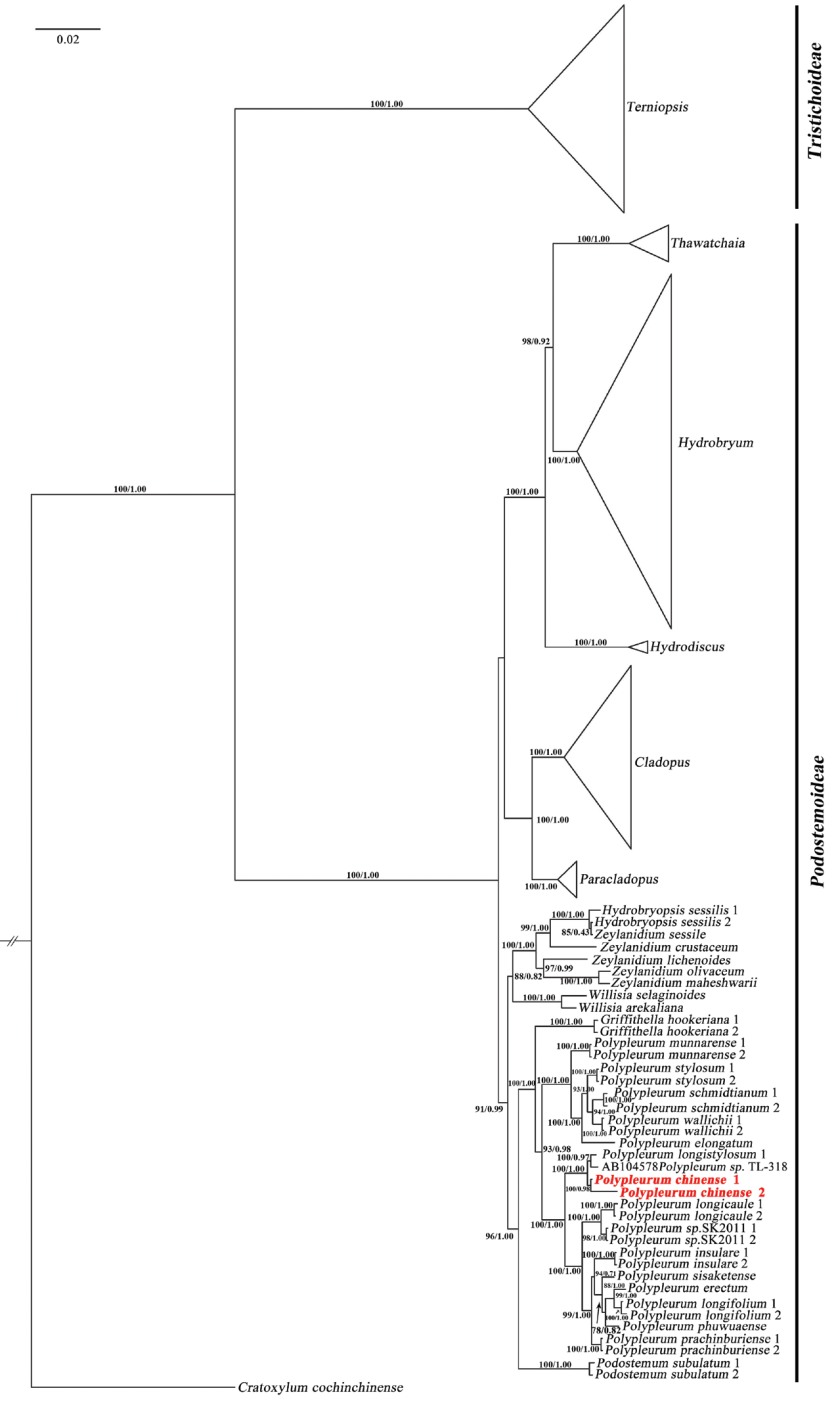
Phylogenetic tree of Asian Podostemaceae, based on Bayesian Inference of *matK* sequences. Numbers above and below branches indicate RAxML (left) bootstrap probabilities (BP) and Bayesian (right) posterior probabilities (PP), respectively. Triangles indicate clades containing multiple species (samples) of one genus examined and the vertical lengths of triangles reflect the number of species (samples) examined.

**Figure 8.**
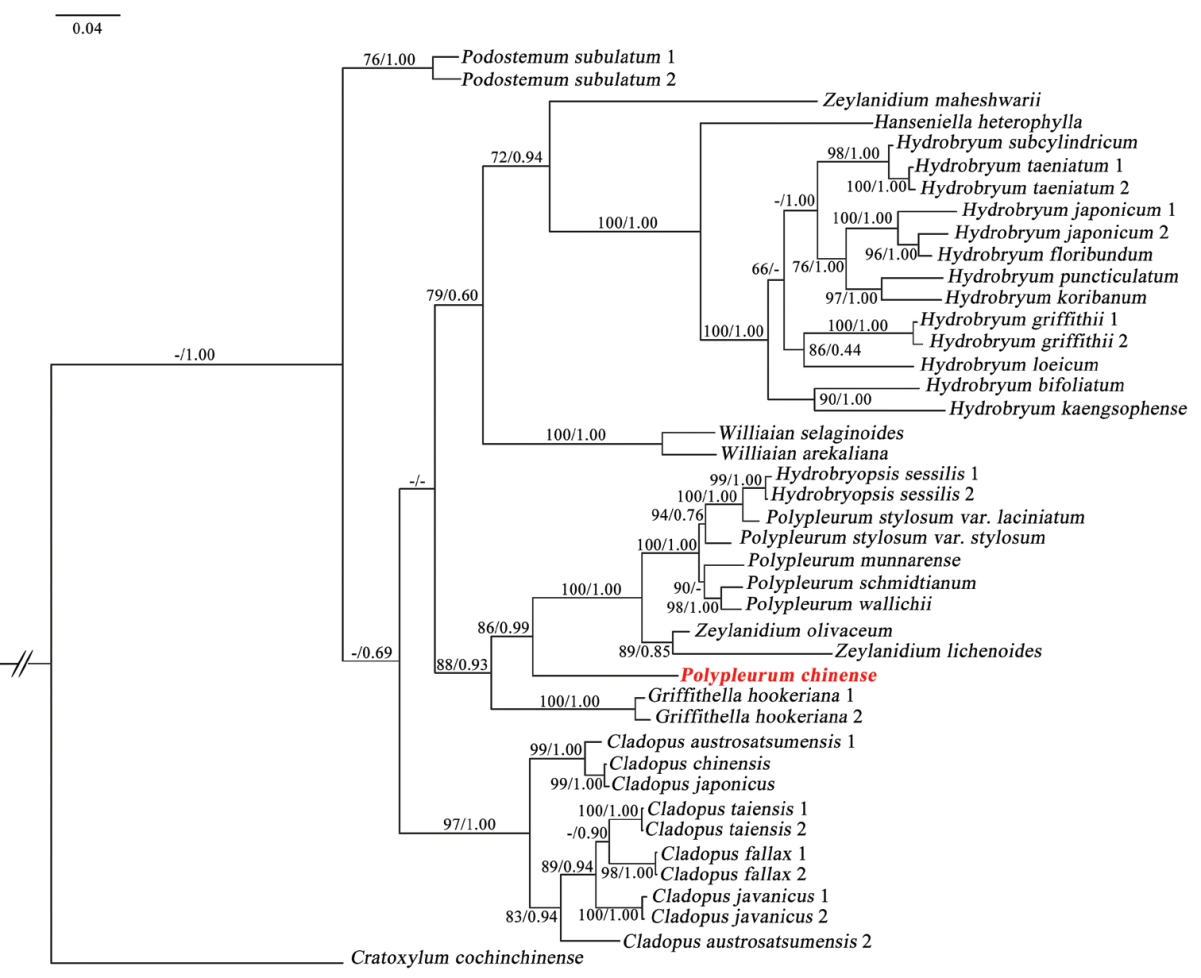
Phylogenetic tree of Asian Podostemaceae, based on Bayesian Inference of nrITS sequences. Numbers above and below branches indicate RAxML (left) bootstrap probabilities (BP) and Bayesian (right) posterior probabilities (PP), respectively.

Phylogenies were reconstructed by the Maximum Likelihood (ML) and Bayesian Inference (BI) analyses using the *matK* and nrITS sequences. The phylogenetic study, based on *matK* sequences suggested the two subfamilies, Tristichoideae and Podostemoideae are sister groups. *Polypleurum* is a monophyletic group within Podostemoideae, which is divided into two subclades. *Polypleurum* is closer to *Griffithella*. *Polypleurumchinense* is sister to *P.longistylosum* with strong support (PP = 1, BS = 100) and nested in a clade formed by nine other species of *Polypleurum*. *Polypleurumchinense* 1 is extracted from the complete chloroplast genome and *P.chinense* 2 is a cloned *matK* sequence (Fig. 7). The phylogenetic analysis, based on the nrITS sequences, suggested that *P.chinense* is sister to a clade formed by *Hydrobryopsissessilis*, *P.stylosum*, *P.schmiditianum*, *P.wallichii*, *P.munnarense*, *Zeylanidiumlichenoides* and *Z.olivaceum* with strong support (PP = 0.99, BS = 86%) (Fig. 8).

## ﻿Discussion

### ﻿Morphology

Amongst the 17 known species of *Polypleurum*, only two species, i.e. *P.longistylosum and P.schmidtianum*, have a single stamen and the shoots or tufts of leaves borne on both sides of the root between the root branches. Although *P.chinense* has an overall morphology similar to *P.longistylosum*, there are some obvious differences, such as narrower roots (0.6–0.8 mm vs.1.0–1.5 mm), leaves appearing in tufts of 8–12 (vs. 4–8) and being as long as 23.1 mm (vs. 5 mm) long; fewer bracts (4 vs. 6); spathella with short spiny or glandular hairs (vs. papillate) on its coat; capsule with 12–14 conspicuous ribs (vs. 10–12 inconspicuous ribs) (Kato 2006). The morphological differences between *P.chinense* and *P.schmidtianum* are more prominent. In *P.schmiditianum*, the roots are wider (ca. 2–4 mm), the peduncle is much longer (ca. 6–7 mm); the ovary is protruding from the spathella at anthesis, 2-locular; the stigmas are much shorter than the ovary; and the capsule is 8-ribbed (Kato 2006) (Table 1). In addition, the previous study reported that the capsule stalk length of *Polypleurum* species was 4–20 mm (Kato 2006), but the capsule stalk of the new species is less than 2 mm long, which is the shortest of all the known species.

### ﻿Comparative analysis of the plastomes

A comparison of the plastome of *Polypleurumchinense* is made with six other species of Podostemaceae with available data (Table 3). The plastome lengths of the seven species varied from 129,074 bp (*Terniopsisyongtaiensis*) to 134,912 bp (*Apinagiariedelii*), with *T.yongtaiensis* being the shortest. For the LCS and SSC regions, the extent of length variation between these species is not evident. The number of PCGs in these species is similar to that of most angiosperms, according to a comparative analysis of gene content (Jin et al. 2020b). The numbers of tRNA and rRNA genes, as well as the GC content, are substantially conserved in all of these plastomes, as shown by our findings. The structure of the IR greatly influences the structural integrity of the entire genome. In all compared species, the *ycf1* and *ycf2* genes, which are two giant open reading frames found in most higher plants, are lost, resulting in a significant reduction of IR regions, hence reducing the size of their plastomes. The loss of *ycf1* and *ycf2* genes were also found in the plastome of Poaceae (Guisinger et al. 2010), Geraniaceae (Weng et al. 2014) and Ericaceae (Braukmann et al. 2017). The functions of *ycf1* and *ycf*2 genes are still controversial and they have not been classified as genes involved in the genetic and photosynthetic systems (Drescher et al. 2000). The size of the IR regions varied amongst compared species, largely due to the evolutionary transfer of complete genes from the SSC regions into the IR or vice versa (Chumley et al. 2006; Wicke et al. 2011).

**Table 3.** Statistics on the basic features of the plastid genomes of *Polypleurumchinense* and related taxa.

Species	Voucher	Accession no.	Length (bp)	LSC (bp)	SSC (bp)	IR(bp)	GC content (%)	No. of PCGs	No. of tRNA	No. of rRNA
*Polypleurumchinense*	CBH 04407	OL944404	132,110	79,022 (~ 59.8%)	12,310 (~ 9.3%)	20,389 × 2(~ 30.9%)	34.85	74	30	4
*Apinagiariedelii*	C.P. Bove 2513 (R)	MN165812	134,912	85,377 (~ 61.0%)	12,437 (~ 8.9%)	21,049 × 2 (~ 30.1%)	34.90	74	30	4
*Marathrumutile*	AMB 497 (ANDES)	MN165814	131,951	79,778 (~ 60.5%)	12,283 (~ 9.3%)	19,945 × 2 (~ 30.2%)	35.10	73	29	4
*Marathrumcapillaceum*	C.P. Bove 2493 (R)	MN165813	134,374	79,990 (~ 59.5%)	12,302 (~ 9.2%)	21,041 × 2 (~ 31.3%)	35.00	75	30	4
*Marathrumfoeniculaceum*	W. D. Stevens - 32072	MK995178	131,600	79,506 (~ 60.4%)	12,262(~ 9.3%)	19,916×2 (~ 30.3%)	35.10	76	30	4
*Tristichatrifaria*	A. Mesterhazy MLI 128(Z)	MN165816	130,285	78,925 (~ 60.6%)	12,662 (~ 9.7%)	19,349 × 2 (~ 29.7%)	36.40	74	30	4
*Terniopsisyongtaiensis*	CBH 04587	OM717943	129,074	79,000 (~ 61.2%)	13,066 (~ 10.1%)	18,504 × 2 (~ 28.7%)	36.20	72	30	4

In *Polypleurumchinense*, *Tristichatrifaria* and *Terniopsisyongtaiensis*, the *rps15 gene* is found at the SSC/IR border, but it is shifted to IRs in *Apinagiariedelli*, *Marathrumutile*, *M.capillaceum* and *M.foeniculaceum* due to the expansion at the IR/SSC boundary. Jin et al. (2020b) found that the relocation of *rps15* gene in *M.foeniculaceum* did not accumulate significant mutations, either because it occurred recently or because the substitution rate was too low to detect. In *P.chinense*, the *trnG-UCC* gene mutates to *trnT-CGU* and, in *M.capillaceum*, it is lost. In addition, it is found that all the species compared have a gene inversion from *trnK-UUU to rbcL*in the LSC region and the size of the inversions for each species is similar (ranging from 51 kb for *P.chinense* to 52 kb for *A.riedelli*). It represents an essential mechanism for plastome rearrangements (Mower and Vickrey 2018). The *rpl23* gene is lacking in plastomes of the other five compared species, except *T.yongtaiensis* (Zhang et al. 2022). However, it is present in plastomes of non-Podostemaceae species, such as *Bonnetiapaniculata* and *Cratoxylumcochinchinense* (Bedoya et al. 2019; Jin et al. 2020b). We do not know the extent of gene loss amongst other Podostemaceae species in China and future sequencing projects will inevitably offer insights into rates and mechanisms of gene loss in plastid genomes.

### ﻿Phylogenetic analysis

The present study confirmed *Polypleurumchinense* is a new species, based on the phylogenetic analysis of *matK* and nrITS sequences, which indicated that *P.chinense* is related to *P.longistylosum*. The phylogenetic study demonstrated that *matK* sequence performed better for the phylogenetic analysis of *P.chinense*, which was consistent with the previous studies (Koi et al. 2012; Kato et al. 2017). The ability of discrimination between species based on nrITS was comparatively poor (Khanduri et al. 2015). Despite the fact that nrITS performed quite well (79%) in angiosperms, lower discrimination success was reported for Ranunculales (6.7%) and Laurales (14.3%) (China Plant BOL 2011). The inconsistencies in species ascriptions between nrITS may result from hybridisation and introgression or incomplete lineage sorting (Alvarez and Wendel 2003; Rieseberg et al. 2006; Feliner and Rosselló 2007). As a result, utilising plastid DNA markers alone may not be sufficient to discriminate between closely-related species. Furthermore, using a single individual for each species, based on plastid DNA markers, might be deceptive (China Plant BOL 2011).

### ﻿The establishment of the new species

The identification of the new *Polypleurum* brings the total number of genera of Podostemaceae to four in China. The discovery of *Polypleurum* in China not only enriches the angiosperm flora of China, but also provides strong evidence for a close connection between the subtropical flora of Fujian and the tropical flora of Southeast Asia.

### ﻿Key to the genera of Podostemaceae of China

**Table:** 

1	Flowers axillary; spathella absent; tepals 3; stamens 2 or 3	***Terniopsis***
–	Flowers terminal; spathella present; tepals 2; stamens 1 or 2	**2**
2	Leaves of fertile stems digitate, 3–9-segmented, scattered, imbricate; capsule smooth	***Cladopus***
–	Leaves of fertile stems scale-like or needle-like, distichous; capsule ribbed	**3**
3	Stamens 2, forked; leaves of fertile stems scale-like; capsules appressed to roots; bracts boat-shaped, entire; roots foliose, with tufts of linear leaves scattered over dorsal surface	***Hydrobryum***
–	Stamen 1, simple; leaves of fertile stems needle-like, capsules erect; bracts ribbon-like; roots, ribbon-like, with tufts of leaves along both sides of root	***Polypleurum***

## Supplementary Material

XML Treatment for Polypleurum chinense

## Supplementary materials

Supplementary material 1 Appendix Data type Primers, Voucher information (docx. file) Explanation note **Table S1.** Primers used in this study. **Table S2.** Voucher information for the taxa used in the present study (*matK*). **Table S3.** Voucher information for the taxa used in the present study (ITS). https://binary.pensoft.net/file/695768This dataset is made available under the Open Database License (http://opendatacommons.org/licenses/odbl/1.0/). The Open Database License (ODbL) is a license agreement intended to allow users to freely share, modify, and use this Dataset while maintaining this same freedom for others, provided that the original source and author(s) are credited.ing-Hua Chen, Miao Zhang, Kai Zhao, Xiao-Hui Zhang, Chang-Li Ge
