# Supplementary material for: ﻿Polypleurumchinense (Podostemaceae), a new species from Fujian, China, based on morphological and genomic evidence
Source: PhytoKeys. 2022 Jun 13;199:167–86. doi: 10.3897/phytokeys.199.85679 (PMC9848973; doi:10.3897/phytokeys.199.85679)
Supplement: Supplementary material 1 — Appendix [file phytokeys-199-167_article-85679__-s001.doc]

Appendix A. Supplementary data

Table S1. Primers used in this study

| Name | Sequence (5′–3′) | Direction | Source |
| --- | --- | --- | --- |
| ITS 1 | TCCGTAGGTGAACCTGCGG | F | White et al.(1990) |
| ITS 4 | TCCTCCGCTTATTGATATGC | R | White et al.(1990) |
| 3914F | TGGGTTGCTAACTCAAYGG | F | Johnson & Soltis (1994) |
| MK-2rR | GTCGGGATGGAGTAGATAATWTA | R | Koi et al. (2008) |

Table S2.Voucher information for the taxa used in the present study (matK)

| S.no. | Taxa | Vouchers | NCBI accession numbers(matK) |
| --- | --- | --- | --- |
| 1 | *Polypleurum chinense* 1 | FNU0041131 | OM937361.1 |
| 2 | *Polypleurum chinense* 2 | FNU0041131 | OM937362.1 |
| 3 | *Polypleurum munnarense* 1 | TNS:A. K. Pradeep 90004 | AB610267.1 |
| 4 | *Polypleurum munnarense* 2 | TNS:A. K. Pradeep 90007 | AB698371.1 |
| 5 | *Polypleurum stylosum* 1 | TNS:SL-103 | AB698388.1 |
| 6 | *Polypleurum stylosum* 2 | TNS:SL-104 | AB698389.1 |
| 7 | *Polypleurum schmidtianum* 1 | TNS:TKF-106 | AB698384.1 |
| 8 | *Polypleurum schmidtianum* 2 | TNS:TIK-23 | AB698383.1 |
| 9 | *Polypleurum elongatum* |  | AB048376.1 |
| 10 | *Polypleurum wallichii* 1 | TNS:L-03 | AB610470.1 |
| 11 | *Polypleurum wallichii* 2 | TNS:LK-09 | AB610471.1 |
| 12 | *Polypleurum longistylosum* 1 | TNS:TL-1503 | AB610266.1 |
| 13 | *Polypleurum* sp. | TNS:TL-318 | AB104578.1 |
| 14 | *Polypleurum longicaule* 1 | TNS:TL-901 | AB610263.1 |
| 15 | *Polypleurum longicaule* 2 | TNS:TKF-10A | AB698364.1 |
| 16 | *Polypleurum pluricostatum* 1 | TNS:TPK-17 | AB698372.1 |
| 17 | *Polypleurum pluricostatum* 2 | TNS:TPK-21 | AB698375.1 |
| 18 | *Polypleurum insulare* 1 | TNS:TL-1304 | AB698360.1 |
| 19 | *Polypleurum insulare* 2 | TNS:TL-1521 | AB698361.1 |
| 20 | *Polypleurum sisaketense* | TNS:TL-1502 | AB610464.1 |
| 21 | *Polypleurum phuwuaense* | TNS:TL-705 | AB610268.1 |
| 22 | *Polypleurum erectum* | TNS:TL-706 | AB610260.1 |
| 23 | *Polypleurum longifolium* 1 | TNS:TL-707 | AB610264.1 |
| 24 | *Polypleurum longifolium* 2 | TNS:TL-904 | AB610265.1 |
| 25 | *Polypleurum prachinburiense* 1 | TNS:TL-1404 | AB610275.1 |
| 26 | *Polypleurum prachinburiense* 2 | TNS:TL-1612 | AB698381.1 |
| 27 | *Thawatchaia trilobata* 1 | TNS:PW-LA_TWA-333 | LC151406.1 |
| 28 | *Thawatchaia trilobata* 2 | TNS:PW-LA_TWA-281 | LC151405.1 |
| 29 | *Thawatchaia laotica* 1 | TNS:TPK-103 | LC151397.1 |
| 30 | *Thawatchaia laotica* 2 | TNS:PW-LA_CM-13 | LC151396.1 |
| 31 | *Cladopus fukienensis* 1 | TNS:Kato et al. | LC144913.1 |
| 32 | *Cladopus fukienensis* 2 | TNS:Kato et al. | LC144912.1 |
| 33 | *Cladopus austroosumiensis* 1 |  | AB048371.1 |
| 34 | *Cladopus austroosumiensis* 2 | Deng Y-F et al. | AB104560.1 |
| 35 | *Cladopus chinensis* |  | AB179654.1 |
| 36 | *Cladopus nymanii* | ID03 | AB104561.1 |
| 37 | *Cladopus japonicus* |  | HQ331573.1 |
| 38 | *Cladopus taiensis* 1 |  | AB048372.1 |
| 39 | *Cladopus taiensis* 2 |  | LC380602.1 |
| 40 | *Cladopus javanicus* 1 |  | LC380600.1 |
| 41 | *Cladopus javanicus* 2 |  | LC380599.1 |
| 42 | *Cladopus fallax* 1 |  | LC380598.1 |
| 43 | *Cladopus fallax* 2 |  | LC380597.1 |
| 44 | *Cladopus pierrei* 1 | TNS:PW-LA_SK-08 | LC151305.1 |
| 45 | *Cladopus pierrei* 2 | TNS:PW-LA_SK-04 | LC151304.1 |
| 46 | *Cladopus queenslandicus* |  | AB300702.1 |
| 47 | *Paracladopus chanthaburiensis* 1 |  | AB300701.1 |
| 48 | *Paracladopus chanthaburiensis* 2 |  | AB293559.1 |
| 49 | *Paracladopus chiangmaiensis* 1 |  | AB293560.1 |
| 50 | *Paracladopus chiangmaiensis* 2 | TNS:LK-223 | AB611703.1 |
| 51 | *Hydrobryum subcylindricoides* |  | LC384155.1 |
| 52 | *Hydrobryum hapteron* |  | LC384151.1 |
| 53 | *Hydrobryum micrantherum* | TL 306 | AB104576.1 |
| 54 | *Hydrobryum japonicum* 1 | SK Wu & Kita 1 | AB104573.1 |
| 55 | *Hydrobryum japonicum* 2 |  | AB038192.1 |
| 56 | *Hydrobryum puncticulatum* | Yakushima | AB104572.1 |
| 57 | *Hydrobryum floribundum* | SHIBUSHI | AB104571.1 |
| 58 | *Hydrobryum griffithii* 1 | TL429 | AB104570.1 |
| 59 | *Hydrobryum griffithii* 2 | TL205 | AB104569.1 |
| 60 | *Hydrobryum loeicum* | TL210 | AB104566.1 |
| 61 | *Hydrobryum kaengsophense* | TL312 | AB104565.1 |
| 62 | *Hydrobryum bifoliatum* | TL310 | AB104564.1 |
| 63 | *Hydrobryum koribanum* |  | AB048374.1 |
| 64 | *Hydrobryum stellatum* |  | LC422798.1 |
| 65 | *Hydrobryum nakaiense* |  | LC384153.1 |
| 66 | *Hydrobryum clandestinum* |  | LC384148.1 |
| 67 | *Hydrobryum verrucosum* |  | LC422818.1 |
| 68 | *Hydrobryum taeniatum* |  | LC422811.1 |
| 69 | *Hydrobryum ramosum* |  | LC422797.1 |
| 70 | *Hydrobryum tardhuangense* | TNS:PW-LA_TWA201 | LC155829.1 |
| 71 | *Hydrobryum chiangmaiense* | TNS:TK-06 | LC155826.1 |
| 72 | *Hydrobryum somranii* | TNS:PW-LA_NE-40 | LC151371.1 |
| 73 | *Hydrobryum phetchabunense* 1 | TNS:PW-LA_TWA-336 | LC151369.1 |
| 74 | *Hydrobryum phetchabunense* 2 | TNS:PW-LA_TWA-252 | LC151362.1 |
| 75 | *Hydrobryum khaoyaiense* | TNS:PW-LA_SK-10 | LC151345.1 |
| 76 | *Hydrobryum vientianense* 1 | TNS:TPK-28 | AB610249.1 |
| 77 | *Hydrobryum vientianense* 2 | TNS:L-02 | AB610245.1 |
| 78 | *Hydrobryum varium* | TNS:TPK-22 | AB610252.1 |
| 79 | *Hydrobryum phurueanum* | TNS:TPK-16 | AB610251.1 |
| 80 | *Hydrobryum takakioides* | TNS:LK-202 | AB610241.1 |
| 81 | *Hydrodiscus koyamae* 1 | TNS:L-11 | AB537382.1 |
| 82 | *Hydrodiscus koyamae* 2 | TNS:L-06 | AB537381.1 |
| 83 | *Hydrobryopsis sessilis* 1 | TNS:KI-119 | AB698237.1 |
| 84 | *Hydrobryopsis sessilis* 2 | TNS:KI-207 | AB698240.1 |
| 85 | *Willisia selaginoides* | TNS:A. K. Pradeep 90006C | AB698421.1 |
| 86 | *Willisia arekaliana* | TNS:A. K. Pradeep 93196 | AB698418.1 |
| 87 | *Zeylanidium olivaceum* |  | AB038207.1 |
| 88 | *Zeylanidium subulatum* 1 | TNS:KI-219 | AB610477.1 |
| 89 | *Zeylanidium subulatum* 2 |  | AB038202.1 |
| 90 | *Zeylanidium lichenoides* | TNS:TK-04 | AB698425.1 |
| 91 | *Zeylanidium maheshwarii* |  | AB048379.1 |
| 92 | *Zeylanidium crustaceum* |  | LC269079.1 |
| 93 | *Zeylanidium sessile* |  | LC269067.1 |
| 94 | *Griffithellahookeriana* 1 | TNS:KI-213 | AB698235.1 |
| 95 | *Griffithella hookeriana* 2 | TNS:KI-103 | AB698233.1 |
| 96 | *Terniopsis chanthaburiensis* 1 |  | LC625800.1 |
| 97 | *Terniopsis chanthaburiensis* 2 |  | LC384161.1 |
| 98 | *Terniopsis brevis* 1 |  | LC342961.1 |
| 99 | *Terniopsis brevis* 2 |  | LC342884.1 |
| 100 | *Terniopsis heterostaminata* 1 |  | LC342918.1 |
| 101 | *Terniopsis heterostaminata* 2 |  | LC342903.1 |
| 102 | *Terniopsis filiformis* 1 |  | LC384159.1 |
| 103 | *Terniopsis filiformis* 2 |  | LC384156.1 |
| 104 | *Terniopsis vapyensis* |  | LC342923.1 |
| 105 | *Terniopsis minor* 1 |  | LC342893.1 |
| 106 | *Terniopsis minor* 2 |  | LC342891.1 |
| 107 | *Terniopsis ubonensis* 1 |  | AB450500.1 |
| 108 | *Terniopsis ubonensis* 2 |  | AB698146.1 |
| 109 | *Terniopsis malayana* 1 |  | AB450034.1 |
| 110 | *Terniopsis malayana* 2 |  | AB698132.1 |
| 111 | *Terniopsis microstigma* 1 |  | LC342922.1 |
| 112 | *Terniopsis microstigma* 2 |  | LC342919.1 |
| 113 | *Terniopsis sessilis* |  | AB048377.1 |
| 114 | *Cratoxylum cochinchinense* |  | AB233787.1 |

Table S3.Voucher information for the taxa used in the present study (ITS)

| S.no. | Taxon | Vouchers | NCBI accession no. |
| --- | --- | --- | --- |
| 1 | *Polypleurum chinense* |  | OM952207 |
| 2 | *Polypleurum munnarense* |  | KF900102.1 |
| 3 | *Polypleurum schmidtianum* |  | LC380648.1 |
| 4 | *Polypleurum wallichii* |  | LC380651.1 |
| 5 | *Polypleurum stylosum* 1 |  | KF900103.1 |
| 6 | *Polypleurum stylosum* 2 |  | KF900110.1 |
| 7 | *Cladopus austroosumiensis* 1 | Deng YF et al | AB104608.1 |
| 8 | *Cladopus austroosumiensis* 2 | Tokiwa | AB104606.1 |
| 9 | *Cladopus chinensis* | CH02, 05 | AB104602.1 |
| 10 | *Cladopus japonicus* | Yamazaki-Tyugaku | AB104603.1 |
| 11 | *Cladopus taiensis* 1 |  | LC380644.1 |
| 12 | *Cladopus taiensis* 2 |  | LC380643.1 |
| 13 | *Cladopus javanicus* 1 | ID02 | AB104609.1 |
| 14 | *Cladopus javanicus* 2 | ID01 | AB104610.1 |
| 15 | *Cladopus fallax* 1 |  | LC380641.1 |
| 16 | *Cladopus fallax* 2 |  | LC380640.1 |
| 17 | *Hanseniella heterophylla* | TL311 | AB104584.1 |
| 18 | *Hydrobryum subcylindricoides* |  | LC538380.1 |
| 19 | *Hydrobryum japonicum* 1 | SK Wu & Kita 1 | AB104599.1 |
| 20 | *Hydrobryum japonicum* 2 | TL204, 206 | AB104598.1 |
| 21 | *Hydrobryum puncticulatum* | Yakushima | AB104595.1 |
| 22 | *Hydrobryum floribundum* | Shibushi | AB104600.1 |
| 23 | *Hydrobryum griffithii* 1 | SK Wu & Kita 2 | AB104593.1 |
| 24 | *Hydrobryum griffithii* 2 | TL205 | AB104592.1 |
| 25 | *Hydrobryum loeicum* | TL210 | AB104586.1 |
| 26 | *Hydrobryum* kaengsophense | TL312 | AB104585.1 |
| 27 | *Hydrobryum* bifoliatum | TL310 | AB104583.1 |
| 28 | *Hydrobryum* koribanum | JK05 | AB104596.1 |
| 29 | *Hydrobryum* taeniatum 1 |  | LC538383.1 |
| 30 | *Hydrobryum* taeniatum 2 |  | LC538382.1 |
| 31 | *Hydrobryopsis* sessilis 1 |  | KF900083.1 |
| 32 | *Hydrobryopsis* sessilis 2 |  | KF900085.1 |
| 33 | *Willisia* selaginoides |  | KF900115.1 |
| 34 | *Willisia* arekaliana |  | KF900113.1 |
| 35 | *Zeylanidium* olivaceum |  | KF900118.1 |
| 36 | *Zeylanidium* subulatum 1 |  | KF900099.1 |
| 37 | Zeylanidium subulatum 2 |  | KF900097.1 |
| 38 | *Zeylanidium* lichenoides |  | KF900117.1 |
| 39 | *Zeylanidium* maheshwarii |  | MF083140.1 |
| 40 | *Griffithella* hookeriana 1 |  | KF900089.1 |
| 41 | *Griffithella* hookeriana 2 |  | KF900091.1 |
| 42 | *Cratoxylum* cochinchinense | 1536 | MG730462.1 |
